# The complete mitochondrial genome of *Ancherythroculter wangi* and its phylogeny

**DOI:** 10.1080/23802359.2018.1443035

**Published:** 2018-02-24

**Authors:** Yuanchao Zou, Junying Zhang, Meng Xie, Tao Zhang, Qi Deng, Zhengyong Wen

**Affiliations:** a College of Life Sciences, Neijiang Normal University, Conservation and Utilization of Fishes resources in the Upper Reaches of the Yangtze River Key Laboratory of Sichuan Province, Neijiang, China;; b School of Life Sciences, Southwest University, Chongqing, China

**Keywords:** *Ancherythroculter wangi*, mitochondrial genome, phylogenetic analysis

## Abstract

*Ancherythroculter wangi,* is a unique freshwater fish and mainly distributed in the upper stream of Yangtze River and its tributary. In this study, the complete mitochondrial genome of *A. wangi* was first determined. The total length of the complete mitochondrial genome is 16,622 bp, contained 13 protein-coding genes (PCGs), 2 rRNA genes, 22 tRNA genes, one D-loop locus, and an origin of replication on the light-strand (OL). The overall nucleotide composition was 31.19% A, 24.84% T, 27.79% C, 16.18% G, with 56.03% AT, respectively. Phylogenetic analysis both highly supported that *A. wangi* showed a close relationship with *Culter mongolicus* and *A. kurematsui*. These data would contribute to elucidate the evolutionary mechanisms and biogeography of *Ancherythroculter* and is useful for the conservation of genetics and stock evaluation for *A. wangi*.

*Ancherythroculter wangi* (Cypriniformes, Cyprinidae, Culterinae, *Ancherythroculter*), is an important economic freshwater fish, which mainly distributed in the upper Yangtze River and its tributaries (Ding 1994). Although *A. wangi* is popular for its delicious taste and high nutrition, unfortunately, the natural populations of this species declined rapidly in recent years due to environmental pollution, long-term overfishing, and habitat degradation (Yue et al. 2000).

In this study, the complete mitochondrial DNA sequence was first determined by the next generation sequencing (NGS). The specimens were collected from Neijiang, Sichuan province of China (30°16′17.05″N, 104°36′34.87″E) in September 2017, and were stored in Zoological Specimen Museum of Neijiang Normal University (accession number: 20170920BB05). A 30–40 mg fin clip was collected and preserved in 95% ethanol at 4 °C. Total genomic DNA was extracted from these caudal fins by a Tissue DNA Kit (OMEGA E.Z.N.A., Norcross, GA, USA) following the manufacturer’s protocol. Subsequently, the genomic DNA was sequenced using the NGS, and then the mitogenome was assembled using *A. nigrocauda* as reference.

The complete mitochondrial genome of *A. wangi* was a circular molecule with 16,622 bp in length (GenBank Accession numberMG 783573). The mitogenome of *A. wangi* contained 2 rRNA genes, 13 protein-coding genes (PCGs), 22 tRNA genes, a D-loop locus, and an origin of replication on the light-strand (OL), which was identical to the other typical vertebrates (Chen et al. 2016; Wang et al. 2017). Most of the genes were encoded on H-strand except for *ND6* and 8 *tRNA* genes. The overall nucleotide composition was 31.19% A, 24.84% T, 27.79% C, 16.18% G, with a slight AT bias of 56.03%. All PCGs initiation codons were ATG, except for *COI* that began with GTG. Correspondingly, 11 PCGs stopped with the complete termination codon TAG, while the rest of PCGs ended with an incomplete termination codon T–– (*COII*, *Cyt b*), which was a little different from *A. nigrocauda* (*ND1* with TAA) and *A. lini* (*ND1* with TAA, *ND2* and *ND3* with T––, *ND4*, *ATP6*, and *COIII* with TA–) (Wan et al. 2013; Chen et al. 2016). Moreover, the 22 tRNA genes ranged in size from 68 bp (*tRNA^Cys^*) to 76 bp (*tRNA^Leu^*, *tRNA^Lys^*). The 12S rRNA and 16S rRNA were 963 and 1691 bp, respectively. Additionally, the OL (31 bp) was located between *tRNA^Asn^* and *tRNA^Cys^*. Furthermore, the D-loop (938 bp) was located between *tRNA^Pro^* and *tRNA^Phe^*.

To confirm the phylogenetic relationships between *A. wangi* and other Culterinae subfamily fishes, phylogenetic analysis were performed on the concatenated dataset of 13 PCGs at nucleotide level with neighbour-joining (NJ) and maximum likelihood (ML) methods (Zou et al. 2017). The tree topologies produced by NJ had nearly a same topology as that of ML tree (Figure 1). *Channa argus* (Channoidei, Channidae) was defined as an outgroup. The other 12 species were divided into two clades. *Hemiculterella sauvagei*, *H. bleekeri*, *H. leucisculus* were clustered into clade B, and the rest of species were clustered into clade A. *A. wangi*, *Culter mongolicus*, and *A. kurematsui* were grouped in one clade, suggested the close relationship of these species, and further confirmed that *A. wangi* belongs to the subfamily Culterinae. In most cases, the same genus was closer but *Sinibrama taeniatus* has a closer relationship with *Megalobrama pellegrini* than *S. macrops.* This situation shows that *Sinibrama* and *Megalobrama* may have descended from the same ancestor.

**Figure 1.**
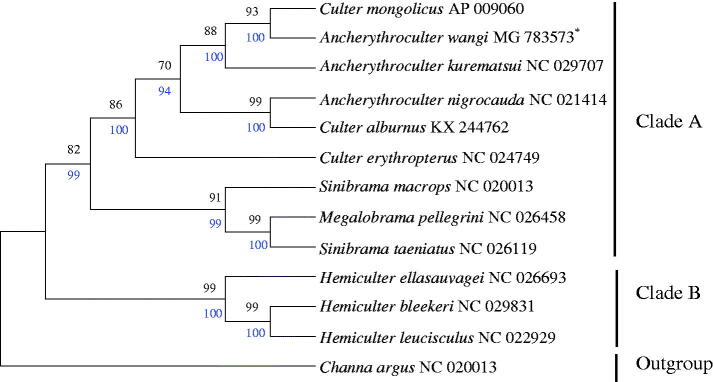
Maximum-likelihood (ML) and neighbour-joining (NJ) phylogenetic tree of *A. wangi* and 12 other species using *C. argus* as an outgroup. Black number and blue number above each node indicates the NJ posterior probability ML bootstrap support values.
